# Supplementary material for: The Mechanisms Involved in the Fluoroquinolone Resistance of Salmonella enterica Strains Isolated from Humans in Poland, 2018–2019: The Prediction of Antimicrobial Genes by In Silico Whole-Genome Sequencing
Source: Pathogens. 2023 Jan 27;12(2):193. doi: 10.3390/pathogens12020193 (PMC9966331; doi:10.3390/pathogens12020193)
Supplement: Supplementary file 1 [file pathogens-12-00193-s001.zip › pathogens-2125399-supplementary.pdf]

Table S1. The results of ciprofloxacin minimum inhibitory concentration, MLST type and resistance genotype by WGS for clinical ciprofloxacin-resistant *S*. Typhimurium

Isolate ID	Year of isolation	Patient status	MLST type	CIP MIC (mg/L)	Fluroquinolone resistance mechanisms				Other AMR gene
					QRDR mutations in:			PMQRs	
					GyrA		ParC		
					Ser 83	Asp 87	Thr 57		
270/19	2019	patient	19	0.38	-	-	-	*qnr*B19	*aac*(6')-Iaa
383/19	2019	patient	19	0.25	Tyr	-	-	-	bla_{TEM-1A}, CARB-2, *sul*1, *tet*(G), *flo*R, *aad*A2, *aac*(6')-Iaa
138/18	2018	convalescent	19	0.19	-	Asn	-	-	CARB-2, *sul*1, *tet*(G), *flo*R, *aad*A2, *aac*(6')-Iaa
29/19	2019	ND	19	0.19	-	Asn	-	-	CARB-2, *sul*1, *tet*(G), *flo*R, *aad*A2, *aac*(6')-Iaa
94/19	2019	patient	19	0.19	-	Asn	-	-	CMY-2, CARB-2, *sul*1, *tet*(G), *flo*R, *aad*A2, *aac*(6')-Iaa
186/18	2018	patient	19	0.125	-	Asn	-	-	*sul*1, *aad*A2, *aac*(6')-Iaa
297/18	2018	patient	19	0.125	-	Asn	-	-	CARB-2, *sul*1, *tet*(G), *flo*R, *aad*A2, *aac*(6')-Iaa
310/18	2018	patient	19	0.125	-	Asn	-	-	*aac*(6')-Iaa
358/18	2018	patient	19	0.125	-	Asn	-	-	CARB-2, *sul*1, *tet*(G), *flo*R, *aad*A2, *aac*(6')-Iaa
227/19	2019	patient	19	0.125	-	Asn	-	-	CMY-2, *sul*1, *tet*(G), *flo*R, *aad*A2, *aac*(6')-Iaa
350/19	2019	patient	19	0.125	-	Asn	-	-	CARB-2, *sul*1, *tet*(G), *flo*R, *aad*A2, *aac*(6')-Iaa
445/19	2019	patient	19	0.125	-	Asn	-	-	bla_{TEM-1A}, CARB-2, *sul*1, *tet*(G), *flo*R, *aad*A2, *aac*(6')-Iaa

MIC – minimum inhibitory concentration; CIP – ciprofloxacin; PMQR – plasmid-mediated quinolone resistance; ND- not detected; AMR – antimicrobial resistance;

Table S2. The results of ciprofloxacin minimum inhibitory concentration, MLST type and resistance genotype by WGS for clinical ciprofloxacin-resistant monophasic *S*. Typhimurium 1,4,[5],12:i:-

Isolate ID	Year of isolation	Patient status	MLST type	CIP MIC (mg/L)	Fluroquinolone resistance mechanisms				Other AMR gene
					QRDR mutations in:			PMQRs	
					GyrA		ParC		
					Ser 83	Asp 87	Thr 57		
531/19	2019	patient	34	0.5	-	-	-	*qnr*B19	*bla*$_{\text{TEM-1B}}$, *sul*2, *aph*(6)-Id, *aph*(3")-Ib, *aac*(6')-Iaa
83/18	2018	patient	34	0.25	-	-	-	*qnr*B19	*bla*$_{\text{TEM-1B}}$, *sul*2, *tet*(B), *aph*(6)-Id, *aph*(3")-Ib, *aac*(6')-Iaa
463/18	2018	convalescent	34	0.25	Tyr	-	-	-	*bla*$_{\text{TEM-1B}}$, *sul*2, *sul3*, *tet*(B), *cml*A1, *aad*A1, *aad*A2, *ant*(3")-Ia, *aph*(6)-Id, *aph*(3")-Ib, *aac*(6')-Iaa
125/18	2018	patient	34	0.38	-	-	-	*qnr*B19	*bla*$_{\text{TEM-1B}}$, *sul*2, *tet*(B), *aph*(6)-Id, *aph*(3")-Ib, *aac*(6')-Iaa
408/18	2018	patient	34	0.38	-	-	-	*qnr*B19, *qnr*B36	*bla*$_{\text{TEM-1B}}$, *sul*2, *aph*(6)-Id, *aph*(3")-Ib, *aac*(6')-Iaa
450/18	2018	convalescent	34	0.38	Tyr	-	-	-	*sul*2, *sul3*, *tet*(B), *cmL*A1, *aad*A2, *ant*(3")-Ia, *aph*(6)-Id, *aph*(3")-Ib, *aac*(6')-Iaa

MIC – minimum inhibitory concentration; CIP – ciprofloxacin; PMQR – plasmid-mediated quinolone resistance; AMR – antimicrobial resistance;

Table S3. The results of ciprofloxacin minimum inhibitory concentration (MIC), MLST type and resistance genotype by WGS for clinical ciprofloxacin-resistant *S.* Hadar

Isolate ID	Year of isolation	Patient status	MLST type	CIP MIC (mg/L)	Fluroquinolone resistance mechanisms				Other AMR gene
					QRDR mutations in:			PMQRs	
					GyrA		ParC		
					Ser 83	Asp 87	Thr 57		
79/18	2018	patient	33	0.25	-	-	Ser	*qnr*B19, *qnr*B36, *qnr*B82	*tet*(A), *aph*(6)-Id, *aph*(3")-Ib, *aac*(6')-Iaa
86/19	2019	patient	33	0.25	-	-	Ser	*qnr*B19, *qnr*B36	*tet*(A), *aph*(6)-Id, *aph*(3")-Ib, *aac*(6')-Iaa
113/19	2019	patient	33	0.25	-	-	Ser	*qnr*B19	*tet*(A), *aph*(6)-Id, *aph*(3")-Ib, *aac*(6')-Iaa
229/19	2019	patient	33	0.25	-	-	Ser	*qnr*B19	*tet*(A), *aph*(6)-Id, *aph*(3")-Ib, *aac*(6')-Iaa
78/18	2018	patient	33	0.19	-	-	Ser	*qnr*B19, *qnr*B36, *qnr*B82	*tet*(A), *aph*(6)-Id, *aph*(3")-Ib, *aac*(6')-Iaa
92/18	2018	patient	33	0.19	-	-	Ser	*qnr*B19, *qnr*B36, *qnr*B67, *qnr*B82	*tet*(A), *aph*(6)-Id, *aph*(3")-Ib, *aac*(6')-Iaa
197/18	2018	patient	9181	0.19	-	-	Ser	-	*aac*(6')-Iaa
85/19	2019	patient	33	0.19	-	-	Ser	-	*tet*(A), *aph*(6)-Id, *aph*(3")-Ib, *aac*(6')-Iaa
112/19	2019	patient	33	0.19	-	-	Ser	*qnr*B19, *qnr*B67, *qnr*B82	*tet*(A), *aph*(6)-Id, *aph*(3")-Ib, *aac*(6')-Iaa
410/19	2019	convalescent	33	0.19	-	Asn	Ser	-	*tet*(A), *aph*(6)-Id, *aph*(3")-Ib, *aac*(6')-Iaa
411/19	2019	convalescent	33	0.19	-	Asn	Ser	-	*tet*(A), *aph*(6)-Id, *aph*(3")-Ib, *aac*(6')-Iaa
328/19	2019	convalescent	33	0.125	-	Tyr	Ser	-	*tet*(A), *aph*(6)-Id, *aph*(3")-Ib, *aac*(6')-Iaa

MIC – minimum inhibitory concentration; CIP – ciprofloxacin; PMQR – plasmid-mediated quinolone resistance; AMR – antimicrobial resistance;

Table S4. The results of ciprofloxacin minimum inhibitory concentration (MIC), MLST type and resistance genotype by WGS for clinical ciprofloxacin-resistant *S.* Infantis

Isolate ID	Year of isolation	Patient status	MLST type	CIP MIC (mg/L)	Fluroquinolone resistance mechanisms				Other AMR gene
					QRDR mutations in:			PMQRs	
					GyrA		ParC		
					Ser 83	Asp 87	Thr 57		
8/19	2019	patient	32	3	-	Tyr	Ser	*qnr*S1	*bla*$_{TEM-1B}$, *sul*1, *tet*(A), *ant*(3")-Ia, *aac*(6')-Iaa
104/19	2019	patient	32	3	Tyr	-	Ser	*qnr*S1	*bla*$_{TEM-1B}$, *sul*1, *tet*(A), *ant*(3")-Ia, *aac*(6')-Iaa
227/18	2018	convalescent	32	1	-	Tyr	Ser	-	*sul*1, *tet*(A), *ant*(3")-Ia, *aac*(6')-Iaa
165/18	2018	patient	32	0.5	-	Tyr	Ser	-	*sul*1, *tet*(A), *ant*(3")-Ia, *aac*(6')-Iaa
277/18	2018	patient	32	0.5	-	Tyr	Ser	-	*sul*1, *tet*(A), *ant*(3")-Ia, *aac*(6')-Iaa
37/19	2019	carrier	32	0.5	-	Tyr	Ser	-	*sul*1, *tet*(A), *ant*(3")-Ia, *aac*(6')-Iaa
68/19	2019	ND	32	0.5	-	Tyr	Ser	-	*sul*1, *tet*(A), *ant*(3")-Ia, *aac*(6')-Iaa
102/19	2019	patient	32	0.5	Tyr	-	Ser	-	*bla*$_{TEM-1C}$, *sul*1, *sul*2, *tet*(A), *flo*R, *ant*(3")-Ia, *aac*(6')-Iaa
150/19	2019	ND	32	0.5	Tyr	-	Ser	-	*sul*1, *tet*(A), *ant*(3")-Ia, *aac*(6')-Iaa
52/18	2018	patient	32	0.38	-	Tyr	Ser	-	*sul*1, *tet*(A), *ant*(3")-Ia, *aac*(6')-Iaa
69/18	2018	patient	32	0.38	-	Tyr	Ser	-	*sul*1, *tet*(A), *ant*(3")-Ia, *aac*(6')-Iaa
111/18	2018	convalescent	32	0.38	-	Tyr	Ser	-	*sul*1, *tet*(A), *ant*(3")-Ia, *aac*(6')-Iaa
124/18	2018	convalescent	32	0.38	-	Tyr	Ser	-	*sul*1, *tet*(A), *ant*(3")-Ia, *aac*(6')-Iaa
127/18	2018	convalescent	32	0.38	-	Tyr	Ser	-	*sul*1, *tet*(A), *ant*(3")-Ia, *aac*(6')-Iaa
190/18	2018	patient	32	0.38	-	Tyr	Ser	-	*sul*1, *tet*(A), *ant*(3")-Ia, *aac*(6')-Iaa
215/18	2018	patient	32	0.38	-	Tyr	Ser	-	*sul*1, *tet*(A), *ant*(3")-Ia, *aac*(6')-Iaa
271/18	2018	patient	32	0.38	-	Tyr	Ser	-	*sul*1, *tet*(A), *ant*(3")-Ia, *aac*(6')-Iaa
275/18	2018	patient	32	0.38	-	Tyr	Ser	-	*sul*1, *tet*(A), *ant*(3")-Ia, *aac*(6')-Iaa
473/18	2018	patient	32	0.38	-	Tyr	Ser		*sul*1, *tet*(A), *ant*(3")-Ia, *aac*(6')-Iaa
58/19	2019	patient	32	0.38	-	Tyr	Ser	-	*sul*1, *tet*(A), *ant*(3")-Ia, *aac*(6')-Iaa
136/19	2019	ND	32	0.38	Tyr	-	Ser	-	*sul*1, *tet*(A), *ant*(3")-Ia, *aac*(6')-Iaa
137/19	2019	patient	32	0.38	Tyr	-	Ser	-	*sul*1, *tet*(A), *ant*(3")-Ia, *aac*(6')-Iaa
162/19	2019	patient	32	0.38	Tyr	-	Ser	-	*sul*1, *tet*(A), *ant*(3")-Ia, *aac*(6')-Iaa
224/19	2019	patient	32	0.38	Tyr	-	Ser	-	*sul*1, *tet*(A), *ant*(3")-Ia, *aac*(6')-Iaa
343/19	2019	patient	32	0.38	Tyr	-	Ser	-	*sul*1, *tet*(A), *ant*(3")-Ia, *aac*(6')-Iaa

368/19	2019	carrier	32	0.38	Tyr	-	Ser	-	*sul*1, *tet*(A), *ant*(3")-Ia, *aac*(6')-Iaa
518/19	2019	carrier	32	0.38	Tyr	-	Ser	-	*sul*1, *tet*(A), *ant*(3")-Ia, *aac*(6')-Iaa
158/18	2018	patient	32	0.25	-	Tyr	Ser	-	*sul*1, *tet*(A), *ant*(3")-Ia, *aac*(6')-Iaa
181/19	2019	contact	32	0.25	Tyr	-	Ser	-	-
221/19	2019	patient	32	0.25	Tyr	-	Ser	-	*sul*1, *tet*(A), *ant*(3")-Ia, *aac*(6')-Iaa

MIC – minimum inhibitory concentration; CIP – ciprofloxacin; PMQR – plasmid-mediated quinolone resistance; AMR – antimicrobial resistance;

Table S5. The results of ciprofloxacin minimum inhibitory concentration (MIC), MLST type and resistance genotype by WGS for clinical ciprofloxacin-resistant *S.* Newport

Isolate ID	Year of isolation	Patient status	MLST type	CIP MIC (mg/L)	Fluroquinolone resistance mechanisms				Other AMR gene
					QRDR mutations in:			PMQRs	
					GyrA		ParC		
					Ser 83	Asp 87	Thr 57		
93/19	2019	carrier	166	0.75	-	-	Ser	*qnr*B19	*aac*(6')-Iaa
84/19	2019	patient	166	0.38	-	-	Ser	*qnr*B19, *qnr*B36	*aac*(6')-Iaa
116/19	2019	patient	166	0.19	-	-	Ser	*qnr*B19, *qnr*B36	*bla*$_{TEM-1B}$, *tet*(A), *aac*(6')-Iaa
118/19	2019	patient	166	0.19	-	-	Ser	*qnr*B19	*bla*$_{TEM-1B}$, *tet*(A), *aac*(6')-Iaa
382/19	2019	patient	166	0.19	Tyr	-	Ser	-	*sul*2, *tet*(A), *ant*(3'')-Ia, *aac*(6')-Iaa

MIC – minimum inhibitory concentration; CIP – ciprofloxacin; PMQR – plasmid-mediated quinolone resistance; AMR – antimicrobial resistance;

Table S6. The results of ciprofloxacin minimum inhibitory concentration (MIC), MLST type and resistance genotype by WGS for clinical ciprofloxacin-resistant *S.* Virchow

Isolate ID	Year of isolation	Patient status	MLST type	CIP MIC (mg/L)	Fluroquinolone resistance mechanisms				Other AMR gene
					QRDR mutations in:			PMQRs	
					GyrA		ParC		
					Ser 83	Asp 87	Thr 57		
10/19	2019	patient	16	0.25	-	Asn	-	-	*aac*(6')-Iaa
9/19	2019	patient	16	0.19	-	Asn	-	-	*aac*(6')-Iaa
11/18	2018	patient	16	0.125	-	Tyr	-	-	*aac*(6')-Iaa

MIC – minimum inhibitory concentration; CIP – ciprofloxacin; PMQR – plasmid-mediated quinolone resistance; AMR – antimicrobial resistance;

Table S7. The results of ciprofloxacin minimum inhibitory concentration (MIC), MLST type and resistance genotype by WGS for clinical ciprofloxacin-resistant *S.* Enteritidis

Isolate ID	Year of isolation	Patient status	MLST type	CIP MIC (mg/L)	Fluroquinolone resistance mechanisms				Other AMR gene
					QRDR mutations in:			PMQRs	
					GyrA		ParC		
					Ser 83	Asp 87	Thr 57		
232/19									
314/19	2019	convalescent	11	0.5	Tyr	-	-	-	*aac*(6')-Iaa
335/19	2019	convalescent	11	0.5	Tyr	-	-	-	*aac*(6')-Iaa
228/18	2018	patient	11	0.38	Tyr	-	-	-	*aac*(6')-Iaa
248/18	2018	patient	11	0.38	-	-	-	*qnr*B19	*aac*(6')-Iaa
315/18	2018	patient	11	0.38	Tyr	-	-	-	*aac*(6')-Iaa
1/19	2019	patient	11	0.25	Tyr	-	-	-	*aac*(6')-Iaa
18/19	2019	patient	11	0.25	Tyr	-	-	-	*aac*(6')-Iaa
24/19	2019	patient	11	0.25	Tyr	-	-	-	*aac*(6')-Iaa
60/19	2019	patient	11	0.25	Tyr	-	-	-	*aac*(6')-Iaa
105/19	2019	patient	11	0.25	Tyr	-	-	-	*aac*(6')-Iaa
156/19	2019	patient	11	0.25	Tyr	-	-	-	*aac*(6')-Iaa
259/19	2019	patient	11	0.25	Tyr	-	-	-	*aac*(6')-Iaa
102/18	2018	patient	11	0.19	Tyr	-	-	-	*aac*(6')-Iaa
108/18	2018	patient	11	0.19	Tyr	-	-	-	*aac*(6')-Iaa
276/18	2018	patient	11	0.19	Tyr	-	-	-	*aac*(6')-Iaa
242/18	2018	patient	11	0.19	Tyr	-	-	-	*aac*(6')-Iaa
123/19	2019	patient	11	0.19	Tyr	-	-	-	*aac*(6')-Iaa
139/19	2019	patient	11	0.19	Tyr	-	-	-	*aac*(6')-Iaa
167/19	2019	patient	11	0.19	Tyr	-	-	-	*aac*(6')-Iaa
169/19	2019	patient	11	0.19	Tyr	-	-	-	*aac*(6')-Iaa
200/19	2019	patient	11	0.19	Tyr	-	-	-	*aac*(6')-Iaa
274/19	2019	convalescent	11	0.19	Tyr	-	-	-	*aac*(6')-Iaa

286/19	2019	patient	11	0.19	Tyr	-	-	-	*aac*(6')-Iaa
308/19	2019	convalescent	11	0.19	Tyr	-	-	-	*aac*(6')-Iaa
318/19	2019	convalescent	11	0.19	Tyr	-	-	-	*aac*(6')-Iaa
324/19	2019	convalescent	11	0.19	-	Tyr	-	-	*aac*(6')-Iaa
357/19	2019	patient	11	0.19	Tyr	-	-	-	*aac*(6')-Iaa
358/19	2019	patient	11	0.19	Tyr	-	-	-	*aac*(6')-Iaa
353/19	2019	patient	11	0.125	Tyr	-	-	-	*aac*(6')-Iaa
355/19	2019	patient	11	0.125	Tyr	-	-	-	*aac*(6')-Iaa
364/19	2019	patient	11	0.125	Tyr	-	-	-	*aac*(6')-Iaa

MIC – minimum inhibitory concentration; CIP – ciprofloxacin; PMQR – plasmid-mediated quinolone resistance; AMR – antimicrobial resistance;
